# Clinical outcomes and complications of robotic-assisted spine surgery: using the ExcelsiusGPS system: a single-surgeon case series

**DOI:** 10.1007/s11701-025-02931-z

**Published:** 2025-11-05

**Authors:** Eric Singh, Tyler Cardinal, Kate Stillman, Solomon Jackson, Carson Cable, Long Di, Seth Tigchelaar, Adham Khalafallah, Timur Urakov

**Affiliations:** Department Neurosurgery, University of Miami Miller School of Medicine, Miami, FL USA

**Keywords:** Robot-assisted spine surgery, ExcelsiusGPS, Minimally invasive spine surgery, Pedicle screw accuracy, Spine instrumentation

## Abstract

Robotic spine surgery with the ExcelsiusGPS system aims to enhance pedicle screw accuracy and reduce complications, though outcome data remain limited. This study evaluates the safety, accuracy, and clinical utility of the ExcelsiusGPS platform in a single-surgeon series, focusing on screw precision, symptom improvement, complication rates, and workflow integration. A retrospective review was performed on 85 consecutive ExcelsiusGPS-assisted spine surgeries performed between January 2022 and December 2024. 56 patients met inclusion criteria with ≥ 90 days of follow-up. Data included demographics, surgical details, outcomes, and imaging. Primary endpoints were screw accuracy, symptom improvement, and operative time. Secondary endpoints included complication rates, revisions, and readmissions. Accuracy was assessed intraoperatively using O-arm imaging and Gertzbein-Robbins grading. Wilcoxon signed-rank tests evaluated symptom changes (*p* < .05). Clopper-Pearson exact methods was utilized to calculate 95% confidence intervals. 56 cases involving 517 screws were analyzed. Mean age was 62.2 years; average fusion involved 4.7 levels and 9.2 screws per case. Half were percutaneous, and 16% were revision surgeries. No intraoperative screw complications occurred. The 90-day revision rate was 0%. 30 day complications occurred in 7.2% of cases, including three surgical site infections and one wound dehiscence. Two patients (3.6%) were readmitted. Significant postoperative improvements were observed in leg and back pain (*p* < .01), weakness (*p* < .01), gait (*p* < .01), constipation (*p* < .46), and urinary symptoms (*p* < .01). Three navigation errors occurred, including one robot abort due to severe scoliosis. One-year proximal junctional kyphosis rate was 5.6%. The ExcelsiusGPS system appears safe and effective for robotic-assisted spine surgery, with high pedicle screw accuracy and low complication and revision rates. These findings support its clinical non-inferiority to conventional methods and highlight opportunities for future research in complex deformities and cost-effectiveness.

## Introduction

Robotic assistance represents a recent advancement in spine surgery, with proposed benefits including improved pedicle screw accuracy, reduced complication rates, and decreased radiation exposure to the surgical team [1, 2]. The first commercially available system, SpineAssist, was introduced in 2004, followed by subsequent innovations such as the Globus ExcelsiusGPS in 2017 [3, 4]. 

Compared to earlier robotic platforms, ExcelsiusGPS offers several key advancements, including a rigid robotic arm and an integrated navigation system that enables direct screw placement with real-time intraoperative guidance [3]. This design eliminates the need for K-wires or clamp-based referencing and provides direct feedback during screw insertion, mitigating issues such as skiving. These features allow for a streamlined setup and aim to enhance operative efficiency, accuracy, and safety in spinal instrumentation.

Despite these innovations, robotic-assisted spine surgery remains limited by a steep learning curve and substantial upfront capital investment [5]. While large-scale studies exist for earlier systems such as Mazor and MazorX, peer-reviewed data on the ExcelsiusGPS platform remain sparse [6]. Given the ongoing debate regarding the role of robotics in spine surgery and the current paucity of literature on this platform, we present this single-surgeon case series to further evaluate its clinical utility.

## Materials and methods

A single-surgeon retrospective analysis of robot-assisted spine surgeries was performed under institutional review board approval with patient consent. The Globus ExcelsiusGPS system was utilized in 85 cases between January 2022 and December 2024.

Adequate follow-up was defined by one of two criteria: (1) ≥ 90 days of documented follow-up, or (2) < 90 days of follow-up with no additional clinic visit planned, as documented in the most recent clinical note. In accordance with institutional protocol and the senior author’s standard practice, patients demonstrating full symptom resolution and return to baseline function may be formally discharged prior to 90 days. These patients are not routinely scheduled for further follow-up unless they become newly symptomatic, reflecting principles of resource stewardship and patient-centered care.

Robotic guidance was used for preoperative screw trajectory planning and intraoperative navigation, all with intraoperative cone-beam CT (O-arm). Intraoperative neurophysiological monitoring was used routinely.

Collected variables included patient demographics, surgical details, and clinical outcomes. Primary endpoints were screw placement accuracy, operative time, and symptom improvement (leg and back pain, weakness, constipation, gait instability, urinary incontinence, numbness, and paresthesias). Screw accuracy was assessed using the Gertzbein-Robbins classification, with Grades A and B considered acceptable a priori. Two authors (ES and SJ) independently reviewed postoperative imaging; disagreements were resolved by a third author (TC). Postoperative imaging was not obtained in 31 patients (55.4%), as per the surgeon’s standard practice to reserve imaging for non-routine recoveries. Secondary endpoints included length of stay, revision surgery, instrumentation type, and registration failures.

All statistical analyses were conducted using SPSS version 29. Descriptive statistics were calculated for demographics, surgical characteristics, follow-up duration, hospital stay, complications, and readmissions. Wilcoxon signed-rank tests were used to compare preoperative and postoperative symptoms, given the ordinal nature of symptom documentation in the electronic medical record. A p-value < 0.05 was considered statistically significant. Clopper–Pearson exact methods were used to calculate 95% confidence intervals for postoperative complications and screw accuracy rates based on Gertzbein-Robbins grading.

## Results

29 patients were excluded due to inadequate follow-up, yielding a final cohort of 56 robotic-assisted spine surgery cases involving 517 pedicle screws with sufficient follow-up. All procedures were performed by a single surgeon at a high-volume academic center between January 2022 and December 2024, using the Globus ExcelsiusGPS system. Of these 56 patients, 20 were voluntarily discharged from follow-up prior to 90 days due to full resolution of symptoms.

Regarding surgical positioning, 42.9% of screws were placed in laterally positioned patients, 51.8% in the prone position, and 5.3% in the supine position. Anatomical regions addressed included the cervical spine (1.7%), thoracic spine (28.6%), lumbar spine (85.7%), and sacrum (39.3%). Surgical indications included degenerative pathology (78.6%), spinal deformity (19.6%), and resection of metastatic lesions (1.8%).

Among the screws evaluated by postoperative imaging, 98.8% were classified as Gertzbein-Robbins Grade A or B (95% CI: 96.5%–99.7%), while 1.2% were Grade C (95% CI: 0.24%–3.38%). No screws were graded below C (Table 1). The mean patient age was 62.2 years. On average, 4.7 levels were fused and 9.2 screws were placed per case. Half of the surgeries were performed percutaneously, and 16% were revision procedures. The average operative time was 368 min, mean hospital stay was 5.4 days, and mean follow-up duration was 270 days (Table 2).

**Table 1:** Summary of screw placement

	# of Screws	% of Screws	95% CI
Screws w/Post Operative Imaging	256	49.5	
Gertzbein-Robbins Grade			
A + B	253	98.8	96.5–99.7
C	3	1.2	0.24–3.38
D	0	0	
E	0	0	

Table 4 summarizes the amount of patients who received post operative imaging

CI – Confidence Interval

**Table 2:** Patient and surgical characteristics

Characteristic	Value	SD
No. Of Surgical Cases	56	
Prone Positioned Pt, %	51.8	
Lateral Positioned Pt, %	42.9	
Supine Positioned Patient, %	5.3	
Age (mean)	65.2, 14.2	14.2
ASA score (mean)	2.5	
BMI (mean)	27.8, 5.1	5.1
Levels Operated per Case (mean)	4.7, 2.6	2.6
No. of Screwed Placed Robotically Per Case (mean)	9.2, 4.6	4.6
Instrumentation Technique		
Percutaneous (%)	50	
Open (%)	50	
Primary or Revision Surgery		
Primary %	83.3	
Revision %	16.7	
Follow Up (mean days)	270.1,251	251

Table 1 presents basic demographic and surgical characteristics

No. – Number, Pt – Patient, SD – Standard Deviation

Intraoperative complications occurred in 7.1% of cases, consisting of four dural tears (7.1%, 95% CI: 2.0%–17.3%), unrelated to robotic screw placement. Complications within 30 days were observed in 7.1% (95% CI: 2.0%–17.3%), of patients and included three surgical site infections (5.4%, 95% CI: 1.1%–14.9%), and one case of wound dehiscence (1.8%, 95% CI: 0.045%–9.5%). These four cases had a mean operative time of 414 min, compared to the series average of 368 min. Two patients (3.6%, (95% CI: 0.4%–12.3%), were readmitted within 90 days, one for wound washout and one for resection of a cystic mass of unclear etiology. No patients required surgical revision within the 90-day period (Table 3). Of the 18 patients followed up at one year, only 5.6% developed symptoms of PJK.

**Table 3:** Complications, revisions and readmissions

Variable	Value %	95% CI
Intraoperative Complications	7.1	
Dural Tear	7.1	2.0–17.3.0.3
Implant Related	0	0.0–6.4.0.4
Screw Revisions	0	0.0–6.4.0.4
Post Op Complication	7.1	
Wound Dehiscence	1.8	0.045–9.5.045.5
Surgical Site Infection	5.4	1.1–14.9
Readmission < 90 Days	3.6	0.4–12.3
Surgical Revision < 90 Days	0	0.0–6.4.0.4

Table 2 demonstrates percentages of various tracked complications, readmissions, and revisions

CI – Confidence Interval

The primary surgical goal in all cases was decompression of neural elements and resolution of presenting symptoms. Symptom improvement was assessed using Wilcoxon signed-rank testing. Each symptom was graded based on electronic medical record documentation as follows: very symptomatic (–2), mildly symptomatic (–1), asymptomatic (0), mildly improved (+ 1), or significantly improved/full resolution (+ 2). These values were used to calculate a Wilcoxon Z-statistic and corresponding p-value. The Z-statistic reflects the direction and magnitude of change in symptom severity before and after surgery, based on the distribution of signed ranks. Statistically significant postoperative improvements (presented in bold)  were observed in leg pain, back pain, leg weakness, constipation, gait instability, and urinary incontinence (Table 4).

**Table 4:** Symptom change w/surgery

Symptom	Change w/Surgery (Z, *P*)
Leg Pain	**−6.0**,**<0.001**
Back Pain	**−6.0**,**<0.001**
Weakness	**−3.2**,**0.001**
Constipation	**−2**,**0.046**
Gait Instability	**−3.5**,**<0.001**
Urinary Incontinence	**−3.6**,** < 0.001**
Numbness	−1.5,0.143
Paresthesia	−1.2,0.143

Table 4 demonstrates the Z and P values of changes in clinical symptoms pre and post operatively. Bold values show statistical significance

## Discussion

Spinal instrumentation is a cornerstone in the treatment of myelopathy and radiculopathy in the setting of instability and other degenerative or structural spine pathologies [7]. However, pedicle screw placement remains technically demanding due to the close proximity of critical neural elements. Over time, numerous innovations have aimed to improve screw accuracy, with robotic-assisted spine surgery representing one of the most recent advancements. The ExcelsiusGPS system by Globus, introduced in 2017, offers real-time intraoperative imaging, automatic compensation for patient movement, and immediate feedback in cases of drill skiving or reference frame shifts. Additionally, its rigid robotic arm enables direct screw insertion without the need for K-wires or clamps [3]. These features distinguish it from earlier robotic platforms and support ongoing investigation into its clinical utility.

Despite these innovations, the ExcelsiusGPS platform has limited published outcomes. Only two large studies currently exist: one reporting 97.5% screw accuracy across 326 screws and another reporting 99% accuracy across 636 screws [8, 9]. Smaller case series have demonstrated the robot’s versatility in procedures such as outpatient minimally invasive transforaminal lumbar interbody fusion and sacroiliac joint fusion [10, 11]. It has also been shown to be non-inferior to traditional methods for spinal trauma instrumentation [12], and one recent study reported its use in palliative radiofrequency ablation combined with vertebroplasty or kyphoplasty for spinal metastases.

The 98.8% accuracy rate observed in our series aligns with prior studies and compares favorably with the 95.5% accuracy reported in a systematic review of 4,368 cases using 3D fluoroscopic navigation [13]. This high level of accuracy supports the senior surgeon’s workflow of reserving postoperative imaging for patients with non-routine recovery. Nonetheless, we acknowledge that a selective imaging protocol may underestimate the true rate of malposition. While intraoperative O-arm imaging provides high confidence in trajectory accuracy [14], asymptomatic misplacements may go undetected without routine imaging.

Three cases of misaligned preplanned screw positioning were encountered in this series. In one case, the procedure was converted to freehand due to the complexity of scoliosis anatomy, which exceeded the robot’s capabilities. While other robotic systems have been studied in scoliosis correction, literature specific to ExcelsiusGPS in this context is lacking [15]. In a second case, unanticipated motion of the robotic arm led to loss of alignment, requiring fluoroscopic conversion for one screw. The third case involved a loss of registration mid-procedure, which was resolved with a repeat O-arm spin allowing for completion of the case with robotic assistance. These events highlight the importance of backup strategies and the need for future studies to systematically track navigation failures as a secondary outcome metric.

Four intraoperative dural tears were observed, though none were attributed to screw placement. Two occurred late in the case with unclear etiology; one followed resection of a synovial cyst, and one resulted from dense adhesions near a lumbar burst fracture. All four patients had accurate screw placement on postoperative imaging (Gertzbein-Robbins Grade A or B). Zero screw related intraoperative complications are in line with other large datasets published on the ExcelsiusGPS [8, 9]. 

Although unrelated to hardware, our dural tear rate of 7.4% is at the higher end of published ranges [16]. However, known risk factors including advanced age, revision status, and burst fractures were present in these cases, consistent with the literature [17, 18]. 

Unlike the two larger ExcelsiusGPS studies, which did not report complication or follow-up data [8, 9], our study provides 30- and 90-day outcomes. The 30-day complication rate was 7.2%, consisting of three surgical site infections (5.4%) and one wound dehiscence (1.8%). These events were not related to screw placement, and operative times for these cases (mean 414 min) were longer than the cohort average (368 min), likely due to case complexity—one was a scoliosis revision requiring 528 min. We do not believe that the perioperative antibiotic regimen (vancomycin and cefepime, used in all cases) contributed to infection risk, as this protocol is consistent with standard practice [19]. 

Two patients (3.6%) were readmitted within 90 days—one for wound washout and one for evaluation of a postoperative cystic mass. Although the latter case raised concern for a CSF leak or urine leak, urologic evaluation was inconclusive, and imaging confirmed appropriate screw placement. For comparison, a multicenter study of the Mazor and Mazor X systems reported a 6.6% 90-day surgical revision rate, higher than in our cohort [6]. 

At one-year follow-up, two patients required reoperation due to new neurologic symptoms (hemilaminectomy and decompression/fusion, respectively), but neither was related to hardware malposition. A study showed a 6.15% surgical revision rate for postoperative radiculopathy at one year compared to our 3.6% [20]. Three additional patients were scheduled for surgery due to proximal junctional kyphosis (PJK), screw/hardware loosening without clear medical comorbidity, and adjacent segment disease. Prior studies report one-year PJK rates between 32–35%; [21–23] our observed rate was 5.6% among the 18 patients with one-year follow-up. These lower rates may reflect careful patient selection or benefit from robotic guidance, though longer-term studies are needed. Symptom tracking demonstrated significant postoperative improvements in leg and back pain (*p* <.01), weakness (*p* <.01), gait instability (*p* <.01), urinary symptoms (*p* <.01), and constipation (*p* =.46) (Table 4).

These findings support the use of robotic navigation not only for technical precision but also for reducing early hardware-related complications. With a 0% intraoperative screw revision rate and low 90-day revision rates, robotic assistance may offer added value in complex or multilevel fusion cases, where manual accuracy is more variable. Although our study lacks a control group, many prior studies have shown that robotic screw placement is non-inferior to conventional techniques with comparable patient outcomes and complication rates [24, 25]. 

Future research should investigate the efficacy of ExcelsiusGPS specifically in scoliosis cases. One procedure in this series was converted to freehand screw placement due to excessive curvature, and to date, no dedicated studies have assessed this platform’s performance in spinal deformity correction. Additionally, tracking navigation loss intraoperatively, such as registration failures or trajectory misalignments, could serve as a valuable secondary outcome in future studies, particularly as no existing literature addresses this aspect for the ExcelsiusGPS system.

This study has several limitations. First, it is a retrospective, single-surgeon case series with no control group. Although this design allowed detailed tracking of complications and readmissions, the absence of a comparator arm limits generalizability. Moreover, the senior surgeon typically reserves robotic assistance for procedures involving > 3 levels of fusion, which may skew outcomes favorably due to selective application. Robotic setup time—including screw planning, O-arm spin, and docking—must be balanced against intraoperative efficiency gains, which may vary by case complexity.

Second, while some patients had < 90-day follow-up, these individuals were formally discharged by the senior author based on full symptom resolution, in line with institutional protocol and resource stewardship principles. Nevertheless, this practice may have limited long-term outcome capture for otherwise uncomplicated cases. Additionally, 29 of the 85 patients (34.1%) were excluded due to inadequate follow-up, raising the possibility of attrition bias. While some patients likely did not return due to full recovery, others may have been lost to follow-up due to dissatisfaction or complications, which could bias our results favorably.

Finally, postoperative imaging was not obtained for all screws, as the senior author reserves imaging for non-routine recoveries. Although intraoperative O-arm guidance offers high confidence in screw trajectory, this protocol could have missed asymptomatic screw malpositions. To address this limitation, Clopper–Pearson exact confidence intervals were calculated for screw accuracy and complication rates to better estimate statistical uncertainty and contextualize potential selection bias.

## **Conclusions**

In summary, this single-surgeon case series of robot-assisted spine surgeries using the ExcelsiusGPS system demonstrated a low complication profile, a 0% 90-day revision rate, and significant symptom improvement in appropriately selected patients. These findings add to the growing body of evidence supporting robotic navigation as a safe and effective tool for complex spinal instrumentation.

While promising, broader adoption of robotic spine surgery must consider the increased costs, operative setup time, and institutional resources required for implementation. Prospective, comparative studies across diverse patient populations are warranted to further define the long-term value and optimal use cases of robotic platforms such as the ExcelsiusGPS.

## Data Availability

Data is provided within the mansucript and also available upon request.

## References

[1] Antonacci CL, Zeng F, Block A, Davey A, Makanji HJJoSS (2024) Robotic-assisted spine surgery—a narrative Rev 10(2):305–312, 10.21037/jss-23-40 PMC11224789 38974496

[2] Lee NJ et al (2022) A multicenter study of the 5-year trends in robot-assisted spine surgery outcomes and complications, (in eng). J Spine Surg 8(1):9–20 10.21037/jss-21-102 PMC8990386 35441099

[3] D’Souza M, Gendreau J, Feng A, Kim LH, Ho AL, Veeravagu A (2019) Robotic-Assisted Spine Surgery: History, Efficacy, Cost, And Future Trends, (in eng), Robot Surg, 6:9–23 10.2147/RSRR.S190720 PMC6844237 31807602

[4] Zygourakis CC et al (2018) Technique: open lumbar decompression and fusion with the Excelsius GPS robot, (in eng), Neurosurg Focus, 45VideoSuppl1 p V6 10.3171/2018.7.FocusVid.18123 29963912

[5] Davidar AD, Jiang K, Weber-Levine C, Bhimreddy M, Theodore N (2024) Advancements in Robotic-Assisted spine Surgery, (in eng). Neurosurg Clin N Am 35(2):263–272 10.1016/j.nec.2023.11.005 38423742

[6] Liounakos JI et al (2022) Ninety-day complication, revision, and readmission rates for current-generation robot-assisted thoracolumbar spinal fusion surgery: results of a multicenter case series, (in eng), J Neurosurg Spine 36(5):841–848 10.3171/2021.8.SPINE21330 34826805

[7] Kim DH, Albert TJ (2002) Update on use of instrumentation in lumbar spine disorders, (in eng), Best Pract Res Clin Rheumatol 16(1):123 – 40 10.1053/berh.2002.0210 11987935

[8] Jain D et al (2019) Initial single-institution experience with a novel robotic-navigation system for thoracolumbar pedicle screw and pelvic screw placement with 643 screws. Int J Spine Surg 13(5):459–463 31741833 10.14444/6060 PMC6833964

[9] Kanaly CW, Backes DM, Toossi N, Bucklen B (2023) A retrospective analysis of pedicle screw placement accuracy using the ExcelsiusGPS robotic guidance system: case series, (in eng), Oper Neurosurg (Hagerstown) 24:3:242–247 10.1227/ons.0000000000000498 36454079

[10] Guillotte A, LeBeau G, Alvarado A, Davis J (2023) Feasibility of outpatient robot assisted minimally invasive transforaminal lumbar interbody fusion. N Ame Spine Soc J (NASSJ) 13:100192 10.1016/j.xnsj.2022.100192 PMC9813734 36620079

[11] Wang TY et al (2022) Sacroiliac Joint Fusion Using Robotic Navigation: Technical Note and Case Series, (in eng), Oper Neurosurg (Hagerstown), 23(1):1–7 10.1227/ons.0000000000000179 35726923

[12] Villeneuve LM, Lee B, Cornwell B, Nagarajan M, Smith ZA (2022) Robot-Assisted thoracolumbar fixation after acute spinal trauma: a case Series, (in eng), Cureus, 14(11):e31832 10.7759/cureus.31832 PMC9788792 36579235

[13] Mason A et al (2014) The accuracy of pedicle screw placement using intraoperative image guidance systems, (in eng). J Neurosurg Spine 20(2):196–203 10.3171/2013.11.SPINE13413 24358998

[14] Shafi KA et al (2022) Does robot-assisted navigation influence pedicle screw selection and accuracy in minimally invasive spine surgery? Neurosurg Focus 52(1):E4 10.3171/2021.10.FOCUS21526 34973674

[15] Al-Naseem AO et al (2024) Robot-assisted pedicle screw insertion versus navigation-based and freehand techniques for posterior spinal fusion in scoliosis: a systematic review and meta-analysis, (in eng), Spine Deform, 12(5):1203–1215 10.1007/s43390-024-00879-y PMC11343815 38619784

[16] Milton R, Kalanjiyam GP, Shetty RSAP, Kanna RM (2023) Dural injury following elective spine surgery - a prospective analysis of risk factors, management and complications, (in eng). J Clin Orthop Trauma 41:102172 37483912 10.1016/j.jcot.2023.102172 PMC10362543

[17] OʼNeill KR, Neuman BJ, Peters C, Riew KD (2014) Risk factors for dural tears in the cervical spine, (in eng), Spine (Phila Pa 1976), 39(17):E1015-20 10.1097/BRS.0000000000000416 24859583

[18] Park JK, Park JW, Cho DC, Sung JK (2011) Predictable factors for dural tears in lumbar burst fractures with vertical laminar fractures. J Korean Neurosurg Soc 50(1):11–6 21892398 10.3340/jkns.2011.50.1.11 PMC3159874

[19] Anwar FN et al (2023) Antibiotic use in spine surgery: A narrative review based in principles of antibiotic stewardship, (in eng). N Am Spine Soc J 16:100278 10.1016/j.xnsj.2023.100278 PMC10641566 37965567

[20] Liounakos JI et al (2021) Reduction in complication and revision rates for robotic-guided short-segment lumbar fusion surgery: results of a prospective, multi-center study. J Robotic Surg, 15(5):793–802 10.1007/s11701-020-01165-5 33386533

[21] Kim DK, Kim JY, Kim DY, Rhim SC, Yoon SH (2017) Risk factors of proximal junctional kyphosis after multilevel fusion surgery: more than 2 years Follow-Up Data, (in eng). J Korean Neurosurg Soc 60(2):174–180 10.3340/jkns.2016.0707.014 PMC5365283 28264237

[22] Onafowokan OO et al (2024) Comparative analysis of outcomes in adult spinal deformity patients with proximal junctional kyphosis or failure initially fused to upper versus lower thoracic spine. 13(24):7722 10.3390/jcm13247722 PMC11678210 39768645

[23] Yasuda T et al (2017) Proximal junctional kyphosis in adult spinal deformity with long spinal fusion from T9/T10 to the ilium, 3(2):204–211 10.21037/jss.2017.06.04 PMC5506304 28744501

[24] Gouzoulis MJ et al (2024) Robotic-Assisted Versus Navigation-Assisted Posterior Lumbar Fusion: a National Database Study, (in eng), Spine (Phila Pa 1976), 49(21):1483–1487 10.1097/BRS.0000000000005032 38717329

[25] Karamian BA et al (2023) Clinical outcomes of robotic versus freehand pedicle screw placement after One-to Three-Level lumbar Fusion, (in eng). Global Spine J 13(7):1871–1877 10.1177/21925682211057491 PMC10556914 34873951

